# The Roles of IL-18 in a Realistic Partial Body Irradiation with 5% Bone Marrow Sparing (PBI/BM5) Model

**DOI:** 10.3390/toxics12010005

**Published:** 2023-12-20

**Authors:** Wanchang Cui, Lisa Hull, Alex Zizzo, Li Wang, Bin Lin, Min Zhai, Vidya P. Kumar, Mang Xiao

**Affiliations:** 1 Armed Forces Radiobiology Research Institute, Uniformed Services University of the Health Sciences, Bethesda, MD 20889, USA; 2 The Henry M. Jackson Foundation for the Advancement of Military Medicine, Inc., Bethesda, MD 20817, USA; 3 Department of Pharmacology and Molecular Therapeutics, Uniformed Services University of the Health Sciences, Bethesda, MD 20814, USA; 4 Department of Pathology, Uniformed Services University of the Health Sciences, Bethesda, MD 20814, USA

**Keywords:** ionizing radiation, IL-18, partial body irradiation with 5% bone marrow sparing (PBI/BM5), knockout mice, colony forming assay, complete blood counts, cytokine

## Abstract

IL-18 has been shown to play important roles in response to total body irradiation. However, homogenous total body irradiation is not a realistic model to reflect the radiation exposure in a real nuclear event. To further study the roles of IL-18 in a real nuclear scenario, we developed a mouse partial body irradiation with 5% bone marrow sparing (PBI/BM5) model to mimic the inhomogeneous radiation exposure. We established the dose response curves of PBI/BM5 using different radiation doses ranging from 12 to 16 Gy. Using the PBI/BM5 model, we showed that IL-18 knockout mice were significantly more radiation resistant than the wild-type mice at 14.73 Gy. We further studied the hematopoietic changes using a complete blood count, bone marrow colony-forming assays, and serum cytokine assays on the mice exposed to PBI/BM5 with IL-18BP treatment and wild-type/IL-18 knockout mice. In conclusion, our data suggest that IL-18 plays important roles in mouse survival in a realistic nuclear exposure model, potentially through the IL-18/IFNγ pathway.

## 1. Introduction

Nuclear radiation exposure can be a serious health threat to millions of people in a large-scale nuclear explosion [1]. Exposed victims may develop acute radiation syndromes (ARS) affecting the hematopoietic or bone marrow syndromes, gastrointestinal syndromes, or cardiovascular/central nervous system syndromes based on the absorbed radiation doses and exposed areas [2]. For victims that survive the ARS, they may develop delayed effects of acute radiation exposure (DEARE) [3,4]. 

IL-18, an IL-1 family member, has been shown to play important roles in response to radiation exposure. IL-18 is not only highly elevated in multiple organs including the thymus, spleen, and bone marrow, but also in the serum and/or urine of multiple animal species including mice, minipigs, and non-human primates (NHP) after total body irradiation (TBI) [5,6,7]. It was shown that the administration of the IL-18 binding protein (IL-18BP), a natural antagonist of IL-18, could significantly increase mouse survival after lethal doses of TBI [8]. The exposure to radiation induced distinct serum cytokine changes in a time dependent manner; however, many cytokines contributed to these changes, thus making it difficult to counterbalance the cytokine changes [9]. Further confirming IL-18’s roles in radiation injuries, IL-18 knockout mice were significantly more radiation resistant compared to the wild-type mice exposed to 9.0 Gy TBI, and their radiation resistance may be related to the distinct gut microbiome composition in the IL-18 knockout mice compared to the wild-type mice [10].

As with most radiation research studies, the previous research about IL-18’s roles in response to radiation were carried out using TBI models. However, the TBI model or localized exposure models cannot mimic the large-scale inhomogeneous radiation exposure following a real nuclear incident [11,12]. It was proposed that partial body irradiation with 2.5–7.5% of bone marrow sparing (PBI/BM2.5–7.5) models should be used for studying radiation toxicity and mitigator development [11,12]. 

In a nuclear disaster, victims can be exposed to very high doses of radiation. For example, the Chernobyl cleanup workers received radiation doses ranging up to 16 Gy [13]. Because it is unethical to expose human test subjects to dangers such as radiation, the FDA developed the “Animal Rules” to guide the development of medical countermeasures using just animal data, without the need for clinical trials (https://www.fda.gov/emergency-preparedness-and-response/mcm-regulatory-science/animal-rule-summary, accessed on 14 December 2023). The major endpoint is “generally the enhancement of survival or prevention of major morbidity” according the Animal Rules. Therefore, animal models need to use radiation doses that can cause morbidity and mortality. With the FDA approval of six hematopoietic ARS mitigators, current efforts are being directed to develop medical countermeasures that aim to treat organ injuries associated with >10 Gy radiation doses. 

Here, we continued to study IL-18’s roles in a realistic nuclear exposure model. First, we established a mouse PBI/BM5 model that could cause mortality. Next, we tested whether IL-18 knockout mice had a different radiation sensitivity compared to wild-type mice using the PBI/BM5 model. We also studied the hematopoietic and bone marrow clonogenicity changes in the wild-type and IL-18 knockout mice after PBI/BM5 exposure. Furthermore, we tested whether IL-18BP treatment could increase mouse survival and induce serum cytokine changes after PBI/BM5. Our data provide important insights into IL-18’s roles in a realistic radiation exposure situation and will help develop effective radiation mitigators. 

## 2. Materials and Methods

### 2.1. Ethics Statement

The animals were housed in an Association for Assessment and Accreditation of Laboratory Animal Care (AAALAC)-approved facility at the Uniformed Services University of the Health Sciences (USUHS). All animal study procedures, including housing, irradiation, survival study, and blood/tissue collection, were reviewed and approved by the USUHS Institutional Animal Care and Use Committee (IACUC) and all experiments were performed in accordance with the guidelines and regulations from the USUHS-IACUC and the USUHS Department of Laboratory Animal Resources (DLAR).

### 2.2. Mice and Animal Care

12- to 14-week-old C57BL6 female IL-18 wild-type and knockout mice (Jackson Laboratory, Bar Harbour, ME, USA) were used. Mice were randomly housed in an AAALAC-approved facility at the USUHS. The animal rooms were maintained at 20–26 °C with 30–70% humidity on a 12 h light/dark cycle. Commercial rodent chow (Harlan Teklad Rodent Diet 8604, Inotiv, Indianapolis, IN, USA) was available *ad libitum*, as was acidified water (pH = 2.5–3.0) to control opportunistic infections. PCR genotyping was performed to confirm the IL-18 knockout or wild-type status of each mouse. 

### 2.3. Irradiation

PBI was carried out as previously described [14] at an estimated dose rate of 2.8 Gy/min using an Elekta Infinity^TM^ linear accelerator (LINAC) (4 MV X-ray) (Elekta, Stockholm, Sweden). A detailed dosimetry had been carried out to determine uniformity of the field, inline penumbra, and the radiation dose distribution to the targeted area as well as the spared part of the body with in-run monitoring of the delivered dose. Ionization chambers, alanine dosimeters with traceability to national standards, and mouse phantoms were used to measure radiation doses. The mice were anesthetized during irradiation using isoflurane. For PBI/BM5, the mice were exposed to specified doses of radiation to the whole body except the hind limbs’ fibulae, tibiae, and feet (which constituted about 5% of bone marrow [15,16]). All animals were transferred back to their respective cages for recovery from anesthesia. The mice were provided with free access to food and acidified water, and monitored daily for 14 days (up to three times a day during d3–d10) for body weight loss, ruffled fur, or any behavioral abnormalities. Mice showing signs of moribundity (significant weight loss, difficulty in breathing and moving, inability to stand, etc.) according to predetermined parameters [17] were euthanized immediately via CO_2_ inhalation exposure and cervical dislocation according to the institutional IACUC guidelines.

### 2.4. IL-18BP Treatment

IL-18BP treatment was performed as described previously [8]. Briefly, recombinant human IL-18BPa (rhIL-18BP, Cat No. 119-BP; R&D Systems, Minneapolis, MN, USA) was subcutaneously injected at 2.0 mg/kg at selected time points. Saline was used as a vehicle control in the animal studies. 

### 2.5. Tissue Collection, Peripheral Blood Cell Counts and Clonogenicity Assay

Blood and other organs were collected at necropsy. Peripheral complete blood cell counts (CBC) were performed using a clinical hematology analyzer (Element HT5, Heska Co. Loveland, CO, USA). A clonogenicity assay was performed as described previously [8]. Briefly, bone marrow cells were collected from mouse femurs at tissue collection. After erythrocytes were lysed with Erythrocyte Lysis Buffer (Qiagen GmbH, Hilden, Germany), the total BM myeloid cell viability from each mouse was measured using Trypan blue staining. The clonogenicity of mouse BM cells was quantified in standard semi-solid cultures in triplicates using 1 mL of Methocult GF + system (including SCF, IL-3, IL-6 and erythropoietin) for mouse cells (Cat # 03444, StemCell Technologies, Vancouver, BC, Canada) according to the manufacturer’s instructions. Mouse BM cells from individual animals were seeded at 5 × 10^4^ cells/ dish in 35-cm cell culture dishes (BD Biosciences, Franklin Lakes, NJ, USA). The plates were scored for total colony-forming units (CFUs), colonies of erythroid burst-forming units (BFU-E), granulocyte–macrophage (CFU-GM), and mixed-lineage (CFU-GEMM) after culturing for 8–10 days at 37 °C in 5% CO_2_.

### 2.6. Multi-Plex Cytokine Assay

The prepared serum was analyzed to determine the concentrations of 45 cytokines using the Mouse Cytokine/Chemokine 45-Plex Discovery Assay^®^ Array (MD45) (Eve Technologies, Calgary, AB, Canada). These targets include Eotaxin, Erythropoietin, 6Ckine, Fractalkine, G-CSF, GM-CSF, IFNB1, IFNγ, IL-1α, IL-1β, IL-2, IL-3, IL-4, IL-5, IL-6, IL-7, IL-9, IL-10, IL-11, IL-12p40, IL-12p70, IL-13, IL-15, IL-16, IL-17, IL-20, IP-10, KC, LIF, LIX, MCP-1, MCP-5, M-CSF, MDC, MIG, MIP-1α, MIP-1β, MIP-2, MIP-3α, MIP-3B, RANTES, TARC, TNFα, VEGF-A, and TIMP-1.

### 2.7. IL-18 and IL-18BP ELISA Assays

ELISA kits for mouse IL-18 and IL-18BP were acquired from Abcam (Waltham, MA, USA. catalog # ab216165 and ab254509 respectively). The cytokine levels in serum were determined in duplicates following assay instructions provided by the manufacturer.

### 2.8. Statistical Analysis

Kaplan–Meier analysis was performed for survival analysis, and significance between survival curves was determined through a log-rank test to compare survival among groups post-PBI (*n* = 20/group). Probit analysis was performed using IBM SPSS Statistics version 28.0 (IBM Corp, Armonk, NY, USA) and plotted using Graphpad Prism Software version 9.4.1 (GraphPad Software, San Diego, CA, USA). Principal component analysis (PCA) between groups was analyzed using Graphpad Prism Software. Group differences were analyzed using one-way ANOVA, with Tukey’s multiple comparison tests between groups. The results are presented as means ± standard errors of the mean (SEM). *p* < 0.05 is considered statistically significant.

## 3. Results

### 3.1. Radiation Dose-Response Relationship (DRR) after PBI/BM5

**Figure 1:**
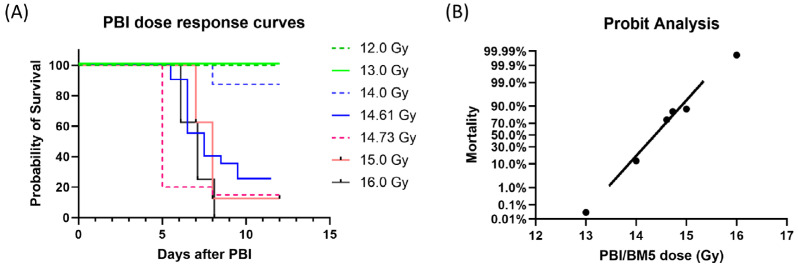
Kaplan–Meier survival curves and probit plot of mice exposed to different doses of PBI/BM5. (**A**) Kaplan–Meier survival curves showing the proportion of mice surviving at each time point for each radiation dose. Each radiation dose is shown using a different color. (**B**) Probit plot showing the DRR of mice exposed to PBI/BM5. Mortality at d12 was analyzed at each dose of radiation and is shown as percent mortality on the Y-axis. Each dot represents one radiation dose. *n* = 8 for the 12.0, 13.0, 14.0, 15.0, and 16.0 Gy groups; *n* = 20 for the 14.61 and 14.73 Gy groups.

Female C57BL6 mice were exposed to different radiation doses of PBI/BM5 and mouse survival was monitored for 12 days. Mice exhibited dose-dependent survival after PBI/BM5 (Figure 1). There was 0% animal death at 12.0 and 13.0 Gy, and 100% animal death at 16.0 Gy as shown on the Kaplan–Meier survival curves. All the animals died during d5 to d10 after radiation exposure (Figure 1A). Figure 1B shows the DRR using probit analysis of C57BL6 exposed to 12–16 Gy PBI/BM5. The LD_30/12_ dose was 14.16 Gy (95% confidence interval, 13.53–14.37 Gy). The LD_50/12_ was 14.38 Gy (95% confidence interval, 13.99–14.54 Gy). The LD_70/12_ was 14.59 Gy (95% confidence interval, 14.39–14.78 Gy). 

### 3.2. IL-18 Knockout Mice Are Significantly More Radiation Resistant Compared to the Wild-Type Mice after 14.73 Gy PBI/BM5

**Figure 2:**
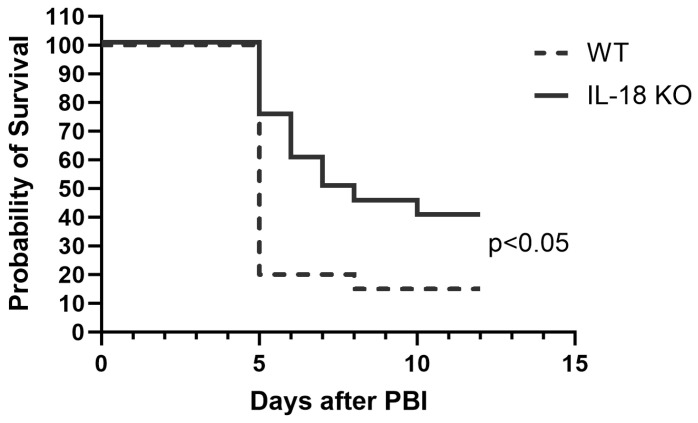
Kaplan–Meier survival curves for the IL-18 knockout and wild-type mice after 14.73 Gy PBI/BM5. *n* = 20 mice for each group. *p* < 0.05 between the two groups.

It was shown that IL-18 knockout mice were significantly more radiation resistant compared to the wild-type mice after total body irradiation [10]. Here we tested whether IL-18 knockout mice behaved similarly in a more realistic radiation model. Female IL-18 knockout and wild-type C57BL6 mice were irradiated with 14.73 Gy PBI/BM5 and their survival was monitored for 12 days. As shown in Figure 2, the IL-18 knockout mice were significantly more radiation resistant compared to the wild-type mice. 15% of the mice survived in the wild-type group and 40% of the mice survived in the IL-18 knockout group. The median survival time was 5.0 days for the wild-type mice and 7.5 days for the IL-18 knockout mice. 

### 3.3. Complete Blood Counts (CBC) of 0 Gy and d14 14.73 Gy in WT and IL-18 KO Mice 

**Figure 3:**
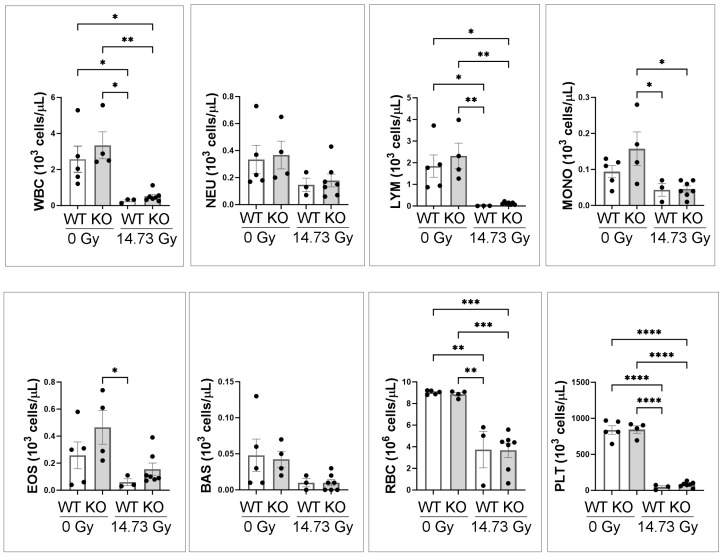
The CBC counts of IL-18 wild-type and knockout mice, 0 Gy controls and at d14 after 14.73 Gy PBI/BM5. Blank bars, WT, wild-type mice; grey bars, KO, IL-18 knockout mice. *n* = 5, 4, 3, and 7 for 0 Gy WT, 0 Gy KO, 14.73 Gy d14 WT, and 14.73 Gy d14 KO mice, respectively. Each dot represents one animal. *, *p* < 0.05, **, *p* < 0.01, ***, *p* < 0.001, ****, *p* < 0.0001.

Peripheral blood counts were performed on the surviving mice at d14 after 14.73 Gy PBI/BM5 and the unirradiated mice. Figure 3 showed the total white blood cell (WBC), neutrophil (NEU), lymphocyte (LYM), monocyte (MONO), eosinophil (EOS), basophil (BAS), red blood cell (RBC), and platelet (PLT) counts. At 0 Gy or d14 14.73 Gy, there was no significant difference between WT and IL-18 KO mice across all blood parameters. WBC, LYM, RBC, and PLT parameters were significantly lower in the d14 14.73 Gy compared to the 0 Gy, irrespective of genotype. NEU and BAS were not significantly different after radiation. 

### 3.4. Bone Marrow Colony Forming Assay of 0 Gy and d14 14.73 Gy in WT and IL-18 KO Mice

**Figure 4:**
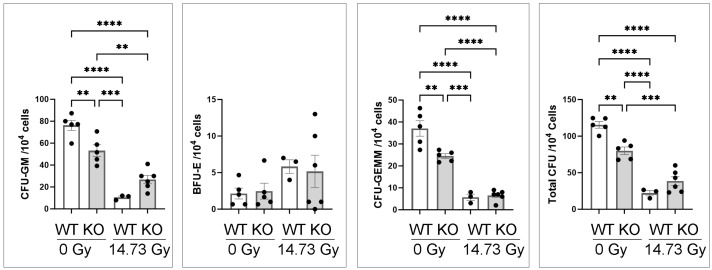
Clonogenicity of mouse bone marrow cells of the IL-18 knockout and wild-type mice, 0 Gy and at d14 after 14.73 Gy PBI/BM5. CFU-GM, BFU-E, CFU-GEMM, and total CFU were counted after 10 days of in vitro culture (*n* = 5, 5, 3, and 6 for 0 Gy WT, 0 Gy KO, 14.73 Gy d14 WT, and 14.73 Gy d14 KO mice, respectively. Each dot represents one animal. Blank bars, WT, wild-type mice; gray bars, KO, IL-18 knockout mice. **, *p* < 0.01, ***, *p* < 0.001, ****, *p* < 0.0001.

We further compared the bone marrow hematopoietic progenitor cells in the IL-18 knockout and wild-type mice at d14 after 14.73 Gy PBI/BM5 and the unirradiated mice as shown in Figure 4. There were significantly more CFU-GM, CFU-GEMM, and total CFU in the 0 Gy mice compared to the irradiated d14 mice irrespective of genotype. Interestingly, 0 Gy WT mice had significantly more CFU-GM, CFU-GEMM, and total CFU compared to the 0 Gy IL-18KO mice. BFU-E was not significantly different among any group. There were no significant differences between WT and IL-18 KO at d14 after 14.73 Gy in the CFU counts.

### 3.5. IL-18BP at 2.0 mg/kg Failed to Rescue C57BL6 Wild-Type Mice after 14.61 Gy PBI/BM5

**Figure 5:**
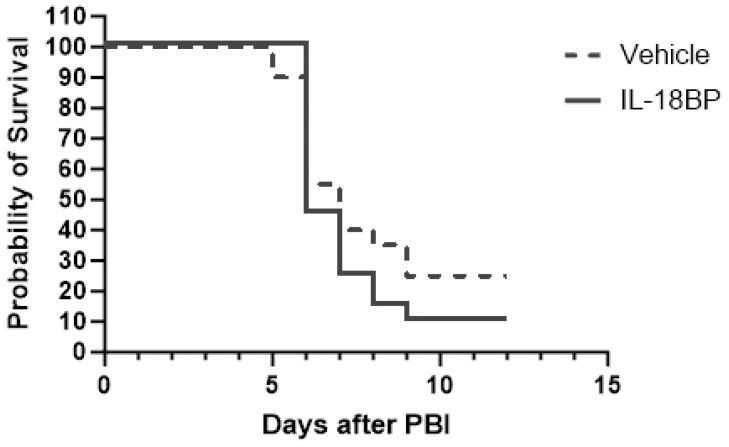
Kaplan–Meier survival curves of mice treated with either a vehicle (saline) or IL-18BP (2.0 mg/kg, on d2 and d5 after radiation) after 14.61 Gy PBI/BM5. *n* = 20 mice for each group. *p* > 0.05 between the two groups.

Because IL-18BP has been shown to significantly improve mouse survival after 9.0 Gy TBI and IL-18 is shown to have a deterministic effect on mouse survival after 14.73 Gy PBI/BM5, we further tested whether IL-18BP treatment could increase mouse survival after a lethal dose of PBI/BM5. C57BL6 female mice were exposed to 14.61 Gy PBI/BM5 and IL-18BP at 2.0 mg/kg was given on d2 and d5 after radiation exposure. Mouse survival was monitored for 12 days after radiation. As shown in Figure 5, IL-18BP at this dose/schedule did not significantly affect mouse survival after 14.61 Gy PBI/BM5. 

### 3.6. Serum Cytokine Changes after PBI/BM5 Using a 45-Plex Assay

**Figure 6:**
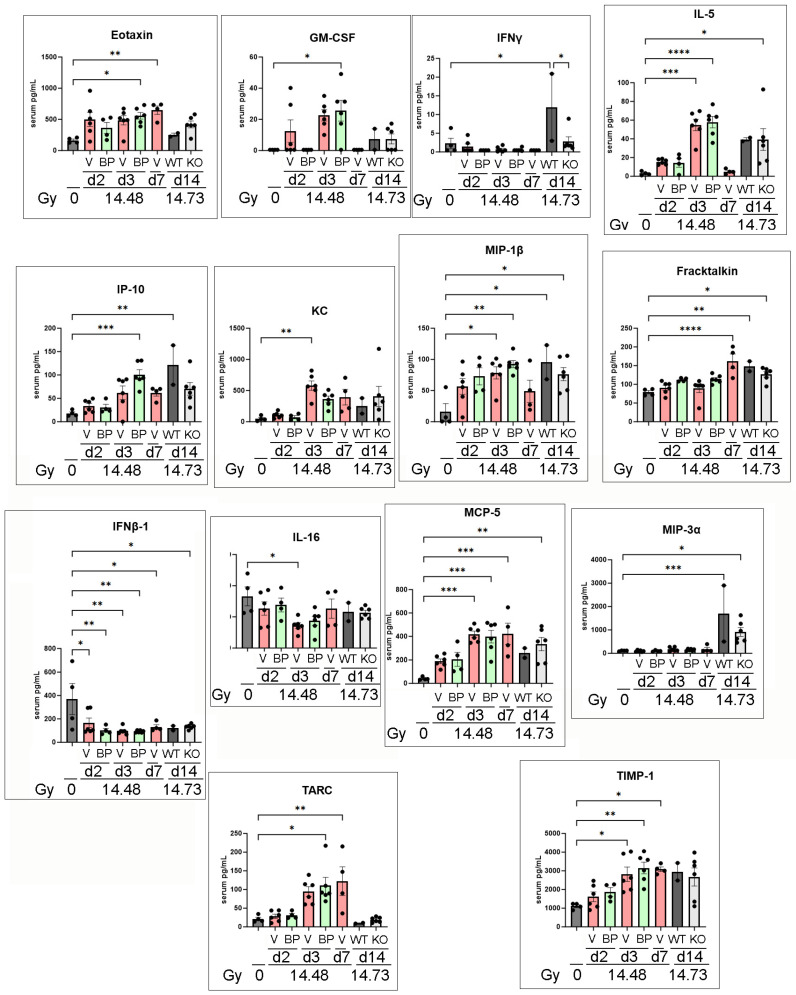
Serum cytokine changes in the C57BL6 mice after PBI/BM5. The mice were exposed to 14.48 Gy PBI/BM5, and then treated with a vehicle or 2.0 mg/kg IL-18BP on d1 after radiation exposure. Serum samples were collected on d2, d3, and d7 after radiation exposure. The serum samples from the IL-18 wild-type and knockout mice were included here too (the d14 serum samples after 14.73 Gy PBI/BM5). A 45-plex cytokine assay was performed on these samples. The cytokine differences between the different groups were analyzed using one-way ANOVA, and a Tukey post-test. To simplify the view, only the significant difference between any group vs. 0 Gy, or the difference between the vehicle and IL-18BP treatment on the same day, or wild-type d14 vs. IL-18KO d14 were shown here. *n* = 4, 6, 4, 6, 6, 4, 2, and 6 mice in the 0 Gy, Veh d2, BP d2, Veh d3, BP d3, Veh d7, WT d14, and IL-18KO d14 groups, respectively. There was only one survivor in the IL-18BP d7 group so no cytokine assay was performed on this animal. V, BP, WT, and KO stand for vehicle group, IL-18BP treatment group, wild-type, and IL-18 knockout. The time of the tissue collection and radiation doses are shown at the bottom. Each dot represents one animal. *, *p* < 0.05; **, *p* < 0.01; ***, *p* < 0.001; ****, *p* < 0.0001.

Even though the IL-18BP treatment did not increase mouse survival after 14.61 Gy PBI/BM5, we suspect that IL-18BP may affect circulating cytokine levels after PBI/BM5. Therefore, we exposed female C57BL6 mice to 14.48 Gy PBI/BM5, and then treated mice with 2.0 mg/kg IL-18BP on d1 after radiation exposure. The mice were euthanized for tissue collection on d2, d3, and d7 after radiation exposure. The samples from this experiment and the serum samples from the IL-18 knockout survival experiment (14.73 Gy from Figure 2) were used for multiplex cytokine assays. Among the 45 cytokines assayed, IL-1β, IL-3, IL-4, IL-7, IL-9, IL-10, IL-12p70, LIF, and VEGF were below detection in all samples and were therefore excluded from further analysis. As shown in Figure 6, multiple cytokines were significantly elevated at multiple time points, particularly MIP-1β, MCP-5, TARC, and TIMP-1 after radiation exposure. IFNβ-1 significantly decreased at multiple time points after radiation exposure. There was no cytokine significantly different between the vehicle and IL-18BP treatment group on d2, d3, or d7. IFNγ was the only cytokine significantly different between the wild-type and IL-18 knockout mice on d14 post PBI/BM5. 

### 3.7. The Serum Levels of IL-18 and IL-18BP in C57BL6 Female Mice after PBI/BM5

**Figure 7:**
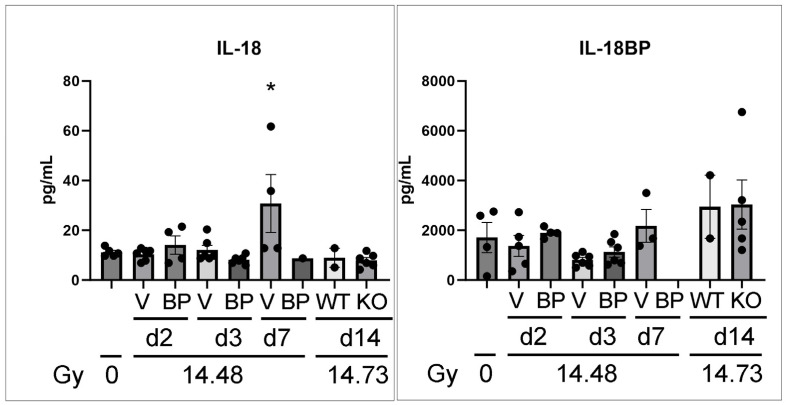
Serum IL-18 and IL-18BP levels in C57BL6 mice after PBI/BM5. The mice were exposed to 14.48 Gy PBI/BM5, and then treated with a vehicle or 2.0 mg/kg IL-18BP on d1 after radiation. Serum samples were collected on d2, d3, and d7 after radiation exposure. The blood samples from the IL-18 WT and KO mice were included here too (d14 blood samples after 14.73 Gy PBI/BM5). The IL-18 and IL-18BP differences between the different groups were analyzed using one-way ANOVA, and a Tukey post-test. The post-test showed no significant difference between the vehicle or IL-18BP treatment on the same day, or wild-type d14 vs. IL-18KO d14. Therefore, the differences shown here were all compared to the 0 Gy group. *n* = 4, 6, 4, 6, 6, 4, 1, 2, and 6 mice in the 0 Gy, Veh d2, BP d2, Veh d3, BP d3, Veh d7, BP d7, WT d14, and IL-18KO d14 groups, respectively. The BP d7 sample was lost in the IL-18BP assay. V, BP, WT, and KO stand for vehicle group, IL-18BP treatment group, wild-type, and IL-18 knockout. The time of the tissue collection and radiation doses are shown at the bottom. Each dot represents one animal. *, *p* < 0.05.

To develop IL-18BP as a radiation mitigator, it is important to know the changes in IL-18 and IL-18BP after radiation exposure. Because the multiplex cytokine assay did not include IL-18 and IL-18BP, we performed individual ELISA assays for these two targets using the same serum samples as those in Figure 6. As shown in Figure 7, serum IL-18 level peaked on d7 after 14.48 Gy PBI/BM5, while the IL-18BP levels were not significantly changed at any time point/treatment/genotype. 

### 3.8. Complete Blood Count Changes after PBI/BM5

**Figure 8:**
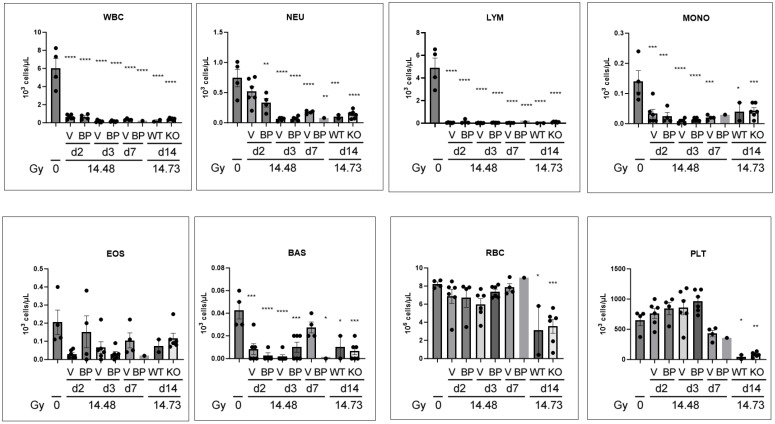
CBC changes in the C57BL6 mice after PBI/BM5. The mice were exposed to 14.48 Gy PBI/BM5, and then treated with a vehicle or 2.0 mg/kg IL-18BP at d1 after radiation. The serum samples were collected on d2, d3, and d7 after radiation. The blood samples from the IL-18 wild-type and knockout mice were included here too (d14 blood samples after 14.73 Gy PBI/BM5). The CBC differences between the different groups were analyzed using one-way ANOVA, and a Tukey post-test. The post-test showed no difference between the vehicle or IL-18BP treatment on the same day, or wild-type d14 vs. IL-18KO d14. Therefore, the differences shown here were all compared to the 0 Gy group. *n* = 4, 6, 4, 6, 6, 4, 1, 2, and 6 mice in the 0 Gy, Veh d2, BP d2, Veh d3, BP d3, Veh d7, BP d7, WT d14, and IL-18KO d14 groups, respectively. V, BP, WT, and KO stand for vehicle group, IL-18BP treatment group, wild-type, and IL-18 knockout. The time of the tissue collection and radiation doses are shown at the bottom. Each dot represents one animal. *, *p* < 0.05; **, *p* < 0.01; ***, *p* < 0.001; ****, *p* < 0.0001.

We also studied the CBC changes after PBI/BM5 using the same animal samples as those in Figure 6. As shown in Figure 8, the WBC, NEU, LYM, MONO, and BAS counts all significantly decreased at multiple time points, regardless of IL-18BP treatment or genotype. EOS counts did not significantly change. The RBC and PLT counts significantly decreased only at the d14 time point. There was no significant difference between the drug treatment groups on the same day, or wild-type vs. IL-18 KO on d14 based on the Tukey post-test. 

### 3.9. Principal Component Analysis (PCA) of Mouse Serum Cytokines and CBC Counts after Irradiation and IL-18BP Treatment

**Figure 9:**
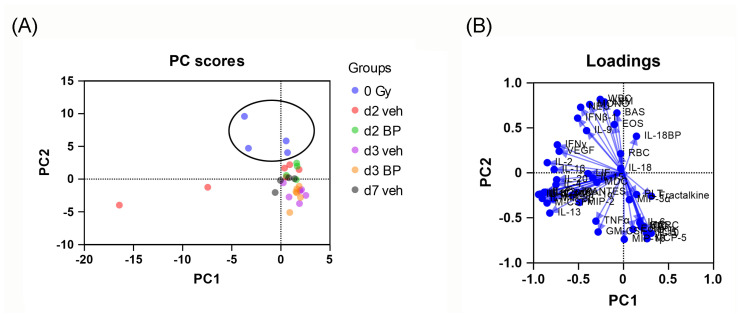
PCA analysis of serum cytokines and CBC in C57BL6 mice with PBI/BM5 and/or IL-18BP treatment. (**A**) PC score plot of non-irradiated mice with d2, d3, and d7 irradiated vehicle/IL-18BP treatment mice. Each dot represents one animal. (**B**) Loading score plot of (**A**). *n* = 4, 6, 4, 6, 6 and 4 for the 0 Gy, d2 vehicle, d2 IL-18BP, d3 vehicle, d3 IL-18BP, and d7 vehicle groups, respectively. The d7 IL-18BP group had only one survivor, and therefore was not included for analysis here.

Because of the availability of a large number of cytokines (around 40 cytokines, including IL-18 and IL-18BP) and CBC parameters, we applied PCA to explore how the cytokines and CBC parameters collectively contribute to patterns of variation in the cytokine/CBC levels across different groups. To simplify the results, we performed PCA analysis in 0 Gy and d2, d3, and d7 vehicle or IL-18BP treatment groups. Distinct divisions were evident between the 0 Gy group and any irradiated groups, as illustrated in Figure 9A, but no separation between the vehicle and IL-18BP treatments on d2 and d3. The loadings represent the coefficients of the linear combination used to construct principal components from the original variables. A variable with a higher loading exerts a more significant influence than variables with smaller loading values. Figure 9B presents the loading values for the PCA analysis. The loading value plots indicate that no individual or small set of variables (such as single or few cytokines or CBC parameters) exerted a pronounced impact in the principal component analysis. Hence, it was necessary to consider multiple variables (i.e., multiple cytokines/CBC parameters) to account for the differences between the groups. 

### 3.10. Individual Mouse Serum Cytokine Changes after 9.0 Gy TBI in CD2F1 Male Mice and 14.48 Gy PBI/BM5 in C57BL6 Male Mice

We compiled the individual mouse serum cytokine changes from a previous study after TBI [9] and the current study of PBI/BM5 (Table 1). The goal was to find similar or different patterns of serum cytokine changes after TBI vs. PBI. For the TBI study, male CD2F1 mice were exposed to 9.0 Gy TBI and blood samples were collected on d3 and d7 after radiation exposure. For the PBI study, female C57BL6 mice were exposed to 14.48 Gy PBI/BM5 and blood samples were collected on d2, d3, and d7 after radiation exposure. Both TBI and PBI showed that there were more significantly increased cytokines than decreased cytokines after radiation exposure. There was a large overlap of the significantly changed cytokines between these two radiation modes. In particular, TARC, IL-5, TIMP-1, MCP-5, and MIP-1β showed significant increases after both TBI and PBI; and IL-16 showed significant decreases after both TBI and PBI. IFNβ1 showed a significant decrease at all three time points after PBI/BM5. 

## 4. Discussion

Radiation exposure models to study radiation toxicity. Traditionally, total body irradiation models are the most widely used models to study radiation toxicity. For example, a PUBMED search in October 2023 retrieved ~19,000 articles using “total body irradiation” as a keyword, and ~2000 articles using “partial body irradiation” as a keyword. In a real nuclear detonation scenario, radiation exposure is generally heterogenous and nonuniform because buildings and other structures can block radiation, particularly the photon component [18,19]. Small amounts of bone marrow spared from the partial shielding can have a fundamental effect on the response to radiation. For example, 5% bone marrow sparing in non-human primates increased the LD_50/60_ radiation dose to 11.01 Gy compared to 7.45 Gy in TBI [15]. 5% bone marrow sparing increased the LD_50/20_ radiation dose to 8.4 Gy compared to 6.5 Gy in TBI in C57BL6 mice, and 10.5 Gy vs. 6.7 Gy in CBA/Ca mice [16]. Localized radiation models used to study an organ injury are also problematic. For example, the thoracic irradiation model does not include the contribution of other organs to lung injury, and thus does not mimick a real radiation exposure effect [11,12,20]. Therefore, realistic radiation models are important for the research and development of medical countermeasures for radiation-induced injuries in a mass casualty scenario [19]. The current consensus is that partial body irradiation with 2.5–7.5% bone marrow sparing (depending on the animal species) models are recommended to study radiation toxicity and medical countermeasure development [11,12]. In the current study, we established a PBI/BM5 model using female C57BL6 mice. This model can be used to study radiation toxicity and test the efficacy of radiation countermeasures. 

IL-18 plays important roles in both the TBI and PBI models. Using transgenic animals, we showed that IL-18 knockout mice were both radiation resistant in a TBI model [10] and the PBI/BM5 model here. Our previous study using TBI showed that IL-18BP could increase mouse survival, potentially through the inhibition of IL-18’s downstream effector IFNγ expression in bone marrow [8]. Similarly, we showed here that IL-18 knockout mice had significantly lower levels of serum IFNγ on d14 after PBI/BM5 compared to the wild-type mice. IL-18 is a well-established inducer of IFNγ production in Th1 and NK cells with or without IL-12 upon stimulation with antigens [21,22]. IFNγ is an important cytokine for inflammatory responses, causing cell death through apoptosis and necrosis [23,24]. Our data suggest the IL-18/IFNγ pathway is important in the response to radiation exposure. Blocking excessive IL-18/IFNγ signaling after radiation exposure may decrease the inflammatory reaction in bone marrow and circulation, thus promoting a faster recovery of bone marrow and other organs. 

Cytokine storms after radiation exposure in the TBI and PBI models. Similar to our reported cytokine storms in mice after TBI [9], our current data here suggest that comparable cytokine storms also happen in mice after PBI/BM5. Many serum cytokines were significantly changed in both the TBI and PBI/BM5 model. Both models showed that no single or few cytokines could explain the difference between non-irradiated and irradiated groups, suggesting a very complex cytokine network after radiation exposure. 

Serum IL-18 changes after TBI and PBI. Our previous study of TBI showed serum IL-18 levels increased on d1 after radiation and continually increased on d3 in a radiation dose-dependent manner [8]. In our current study of PBI/BM5, serum IL-18 levels were only significantly increased on d7 after radiation exposure. The delayed increase of IL-18 may partially explain the inefficacy of IL-18BP treatment against PBI/BM5. The IL-18BP’s half-life was 60.9 h [9]. Therefore, the d2 + d5 injection of IL-18BP could not neutralize the peak levels of IL-18 at d7. A longer treatment schedule of IL-18BP may be effective for PBI/BM5. 

Future studies. The animal deaths during the 5–10 days after radiation exposure are generally linked to acute gastrointestinal injury [16,25]. In the current study, we have focused on the hematopoietic parameters and cytokine changes. In future studies, we will investigate the difference in the intestinal tract of the wild-type and IL-18 knockout mice to explain their radiation sensitivity difference. And we will investigate whether modifying the IL-18BP treatment schedule/doses can mitigate radiation toxicity in the PBI/BM5 model.

## 5. Conclusions

In this manuscript, we successfully established a mouse PBI/BM5 model, which is a realistic model to mimic radiation exposure in a nuclear event. Using this model, we showed that IL-18 plays important roles in response to radiation exposure using transgenic animals. A pharmaceutical blockade of IL-18 needs to be optimized for mitigating radiation-induced toxicity in the current model.

## Figures and Tables

**Table 1:** Changes in individual serum cytokine levels were assessed following 9.0 Gy TBI in CD2F1 male mice and 14.48 Gy in C57BL6 female mice. Serum cytokine levels on days 2, 3, or 7 after irradiation with a vehicle treatment were compared to the 0 Gy controls. All cytokine concentrations were measured in pg/mL. The mean ± standard error of mean (SEM) was presented for each cytokine. Significantly elevated cytokines are represented in red, while significantly decreased cytokines are indicated in green compared to 0 Gy controls. Non-detectable (n.d) and above detection (a.d) are denoted as such, and non-significant changes were labeled as n.s. The TBI data are shown in regular font and PBI data are shown in italic font for better contrast. The study included *n* = 4–6 mice per group, and *p* < 0.05 was considered indicative of a significant change.

	CD2F1 Male Mice Total Body Irradiation 9.0 Gy						C57BL6 Female PBI/BM5 14.48 Gy							
	Cytokines	0 Gy	d3 veh	*p* Value d3 veh vs. 0 Gy	d7 veh	*p* Value d7 veh vs. 0 Gy	Cytokines	0 Gy	d2 veh	*p* Value d2 veh vs. 0 Gy	d3 veh	*p* Value d3 veh vs. 0 Gy	d7 veh	*p* Value d7 veh vs. 0 Gy
1	MCP-1	13.31 ± 1.97	495.87 ± 194.8	*p* < 0.05	138.79 ± 32.75	n.s	*MCP-1*	*40.61 ± 7.4*	*426.73 ± 136.24*	*n.s*	*332.87 ± 32.3*	*n.s*	*394.42 ± 79.67*	*n.s*
2	MDC	325.67 ± 42.59	513.38 ± 72.71	*p* < 0.05	187.58 ± 22.82	n.s	*MDC*	*126.45 ± 21.34*	*352.43 ± 67.67*	*n.s*	*169.01 ± 18.27*	*n.s*	*142.07 ± 42.38*	*n.s*
3	MIP-3β	178.38 ± 24.48	320.74 ± 11.59	*p* < 0.01	283.41 ± 34.49	*p* < 0.05	*MIP-3β*	*87.01 ± 25.42*	*118.62 ± 41.07*	*n.s*	*104.38 ± 20.86*	*n.s*	*114.49 ± 24.46*	*n.s*
4	IL-17	3.12 ± 0.37	8.16 ± 1.56	*p* < 0.01	3.33 ± 0.89	n.s	*IL-17*	*0.64 ± 0.18*	*2.66 ± 1.49*	*n.s*	*0.49 ± 0.12*	*n.s*	*0.52 ± 0.13*	*n.s*
5	MIG	112.85 ± 42.23	690.56 ± 159.29	*p* < 0.01	146.93 ± 29.25	n.s	*MIG*	*90.95 ± 39.63*	*75.87 ± 13.04*	*n.s*	*298.66 ± 31.72*	*n.s*	*158.68 ± 85.08*	*n.s*
6	MIP-1α	46.67 ± 7.3	95.7 ± 10.46	*p* < 0.01	42.61 ± 4.72	n.s	*MIP-1α*	*58.97 ± 28.03*	*80.51 ± 25.82*	*n.s*	*64.78 ± 13.45*	*n.s*	*41.07 ± 14.39*	*n.s*
7	TARC	29.73 ± 3.86	132.74 ± 31.44	*p* < 0.01	43.26 ± 6.11	n.s	*TARC*	*21.1 ± 4.61*	*28.19 ± 5.8*	*n.s*	*94.19 ± 13.2*	*n.s*	*122.08 ± 38.53*	*p < 0.01*
8	IL-5	4.75 ± 1.59	23.37 ± 3.52	*p* < 0.001	18.5 ± 0.94	*p* < 0.01	*IL-5*	*3.01 ± 1.01*	*15.5 ± 1.18*	*n.s*	*54.92 ± 6.1*	*p < 0.001*	*4.96 ± 1.31*	*n.s*
9	IL-1α	23.77 ± 9.04	113.51 ± 17.61	*p* < 0.001	51.19 ± 7.54	n.s	*IL-1α*	*74.28 ± 42.7*	*137.98 ± 61.95*	*n.s*	*80.02 ± 27.54*	*n.s*	*55.62 ± 17.77*	*n.s*
10	TIMP-1	1627.59 ± 330.01	4850.59 ± 707.44	*p* < 0.001	1611.3 ± 190.79	n.s	*TIMP-1*	*1107.43 ± 79.64*	*1608.44 ± 260.94*	*n.s*	*2815.2 ± 384.94*	*p < 0.05*	*3082.38 ± 123.71*	*p < 0.05*
11	TNFα	2.16 ± 0.83	12.25 ± 1.44	*p* < 0.001	2.67 ± 1.56	n.s	*TNFα*	*3.91 ± 2.09*	*5.03 ± 2.33*	*n.s*	*5.92 ± 0.74*	*n.s*	*7.02 ± 2.23*	*n.s*
12	MCP-5	196.13 ± 14.01	1112.96 ± 140.57	*p* < 0.0001	672.18 ± 64	*p* < 0.01	*MCP-5*	*41.17 ± 6.72*	*189.11 ± 20.09*	*n.s*	*418.19 ± 27.08*	*p < 0.001*	*420.98 ± 91.89*	*p < 0.001*
13	G-CSF	378.82 ± 213.87	12031.13 ± 1317.17	*p* < 0.0001	7820.85 ± 1429.67	*p* < 0.001	*G-CSF*	*164.86 ± 57.01*	*850.11 ± 144.38*	*n.s*	*9529.55 ± 2765.24*	*n.s*	*7176.39 ± 2771.2*	*n.s*
14	IFNγ	1.07 ± 0.26	6.88 ± 1.04	*p* < 0.0001	1.45 ± 0.17	n.s	*IFNγ*	*2.26 ± 1.42*	*1.45 ± 0.69*	*n.s*	*0.71 ± 0.29*	*n.s*	*0.46 ± 0*	*n.s*
15	IP-10	66.32 ± 6.74	190.45 ± 19.34	*p* < 0.0001	104.05 ± 10	n.s	*IP-10*	*17.96 ± 3.15*	*33.77 ± 5.44*	*n.s*	*61.96 ± 14.56*	*n.s*	*61.81 ± 7.07*	*n.s*
16	MIP-1β	61.82 ± 10.59	161.69 ± 4.78	*p* < 0.0001	64.12 ± 10.51	n.s	*MIP-1β*	*15.85 ± 13.28*	*56.55 ± 12.59*	*n.s*	*78.45 ± 9.75*	*p < 0.01*	*48.83 ± 17.74*	*n.s*
17	IL-16	1957.48 ± 396.85	935.04 ± 162.28	n.s	429.42 ± 86.09	*p* < 0.01	*IL-16*	*1658.44 ± 304.57*	*1270.86 ± 213.62*	*n.s*	*721.28 ± 89.77*	*p < 0.05*	*1264.56 ± 308.69*	*n.s*
18	MIP-3α	80.5 ± 23.53	206.4 ± 44.29	n.s	343.82 ± 55.68	*p* < 0.01	*MIP-3α*	*106.8 ± 8.48*	*96.87 ± 13.84*	*n.s*	*165.22 ± 35.98*	*n.s*	*177.17 ± 71.92*	*n.s*
19	EPO	246.74 ± 32.09	340.36 ± 30.09	n.s	791.16 ± 110.13	*p* < 0.001	*EPO*	*199.18 ± 59.94*	*224.84 ± 50.66*	*n.s*	*400.07 ± 39.37*	*n.s*	*272.76 ± 23.97*	*n.s*
20	6Ckine/Exodus 2	a.d	a.d	n.s	a.d	n.s	*6Ckine/Exodus 2*	*1285.35 ± 143.2*	*1054.42 ± 117.38*	*n.s*	*1264.96 ± 106.12*	*n.s*	*1236.74 ± 117.84*	*n.s*
21	Eotaxin	826.05 ± 160.13	1472.6 ± 361.65	n.s	1357.4 ± 222.35	n.s	*Eotaxin*	*163.36 ± 20.95*	*499.88 ± 114.34*	*n.s*	*485.57 ± 74.16*	*n.s*	*646.88 ± 67.9*	*p < 0.01*
22	Fractalkine	201.36 ± 19.64	370.76 ± 119.56	n.s	250 ± 43.94	n.s	*Fractalkine*	*78.89 ± 4.51*	*90.77 ± 6.81*	*n.s*	*88.81 ± 10.77*	*n.s*	*161.91 ± 19.03*	*p < 0.001*
23	GM-CSF	3.96 ± 1.67	6.76 ± 3.06	n.s	2.24 ± 1.09	n.s	*GM-CSF*	*0.46 ± 0*	*12.5 ± 7.21*	*n.s*	*22.64 ± 3.6*	*n.s*	*0.46 ± 0*	*n.s*
24	IFNβ-1	138.23 ± 59.63	75.54 ± 9.65	n.s	48.13 ± 1.62	n.s	*IFNβ-1*	*370.04 ± 132.54*	*165.51 ± 41.41*	*p < 0.05*	*97.21 ± 12.76*	*p < 0.01*	*131.16 ± 19.04*	*p < 0.05*
25	IL-10	5.96 ± 1.9	4.3 ± 1.28	n.s	2.63 ± 0.84	n.s	*IL-10*	*n.d*	*n.d*	*n.s*	*n.d*	*n.s*	*n.d*	*n.s*
26	IL-11	25.75 ± 3.47	39.79 ± 11.55	n.s	28.57 ± 19.57	n.s	*IL-11*	*18.23 ± 10.94*	*37.94 ± 22.91*	*n.s*	*18.18 ± 5.24*	*n.s*	*23.12 ± 4.29*	*n.s*
27	IL-12p40	1.52 ± 1.06	2.88 ± 1.18	n.s	2.3 ± 1.26	n.s	*IL-12p40*	*5.51 ± 3.69*	*20.05 ± 7.57*	*n.s*	*3.23 ± 1.38*	*n.s*	*7.34 ± 1.38*	*n.s*
28	IL-12p70	2.47 ± 2.02	2.38 ± 1.09	n.s	0.82 ± 0.37	n.s	*IL-12p70*	*n.d*	*n.d*	*n.s*	*n.d*	*n.s*	*n.d*	*n.s*
29	IL-13	43.27 ± 5.81	48.95 ± 10.74	n.s	48.22 ± 6.14	n.s	*IL-13*	*9.11 ± 4.45*	*23.73 ± 9.97*	*n.s*	*13.45 ± 1.68*	*n.s*	*18.74 ± 2.26*	*n.s*
30	IL-15	3.84 ± 1.58	27.39 ± 13.8	n.s	2.54 ± 0.27	n.s	*IL-15*	*37.19 ± 14.89*	*74.65 ± 35.5*	*n.s*	*29.61 ± 4.68*	*n.s*	*30.46 ± 7.03*	*n.s*
31	IL-1β	3.79 ± 0.45	3.81 ± 0.53	n.s	2.85 ± 0.69	n.s	*IL-1β*	*n.d*	*n.d*	*n.s*	*n.d*	*n.s*	*n.d*	*n.s*
32	IL-2	3.25 ± 0.8	6.16 ± 2.31	n.s	2.04 ± 0.95	n.s	*IL-2*	*20.52 ± 14.99*	*18.81 ± 11.58*	*n.s*	*3.98 ± 1.88*	*n.s*	*11.78 ± 4.75*	*n.s*
33	IL-20	78.37 ± 18.03	81.86 ± 11.61	n.s	67.13 ± 11.64	n.s	*IL-20*	*235.53 ± 107.47*	*273.68 ± 102.07*	*n.s*	*186.93 ± 47.32*	*n.s*	*112.52 ± 42.41*	*n.s*
34	IL-3	n.d	n.d	n.s	n.d	n.s	*IL-3*	*n.d*	*n.d*	*n.s*	*n.d*	*n.s*	*n.d*	*n.s*
35	IL-4	n.d	n.d	n.s	n.d	n.s	*IL-4*	*n.d*	*n.d*	*n.s*	*n.d*	*n.s*	*n.d*	*n.s*
36	IL-6	6.42 ± 3.28	26.44 ± 12.7	n.s	18.32 ± 7.34	n.s	*IL-6*	*7.73 ± 3.22*	*20.36 ± 2.89*	*n.s*	*40.59 ± 9.53*	*n.s*	*17.73 ± 5.25*	*n.s*
37	IL-7	0.51 ± 0.03	1.1 ± 0.64	n.s	0.45 ± 0	n.s	*IL-7*	*n.d*	*n.d*	*n.s*	*n.d*	*n.s*	*n.d*	*n.s*
38	IL-9	6.27 ± 1.33	11.23 ± 3.97	n.s	5.48 ± 2.08	n.s	*IL-9*	*n.d*	*n.d*	*n.s*	*n.d*	*n.s*	*n.d*	*n.s*
39	KC	12.45 ± 4.9	812.75 ± 408.45	n.s	241.88 ± 62.98	n.s	*KC*	*49.89 ± 20.16*	*106.07 ± 19.46*	*n.s*	*580.73 ± 75.11*	*p < 0.01*	*394.36 ± 125.92*	*n.s*
40	LIF	0.73 ± 0.28	0.82 ± 0.31	n.s	0.45 ± 0	n.s	*LIF*	*n.d*	*n.d*	*n.s*	*n.d*	*n.s*	*n.d*	*n.s*
41	M-CSF	7.16 ± 1.83	11.87 ± 1.41	n.s	7.77 ± 1.55	n.s	*M-CSF*	*3.9 ± 1.89*	*12.24 ± 7.2*	*n.s*	*5.93 ± 1.05*	*n.s*	*6.58 ± 1.45*	*n.s*
42	MIP-2	141.3 ± 2.91	147.92 ± 2.69	n.s	138.06 ± 1.44	n.s	*MIP-2*	*168.26 ± 6.1*	*172.87 ± 12.05*	*n.s*	*169.05 ± 5.35*	*n.s*	*192.61 ± 26.72*	*n.s*
43	RANTES	19.32 ± 2.81	21.6 ± 7.15	n.s	22.69 ± 4.77	n.s	*RANTES*	*29.24 ± 16.45*	*58.84 ± 17.25*	*n.s*	*21.6 ± 6.23*	*n.s*	*89.02 ± 30.87*	*n.s*
44	VEGF	n.d	0.86 ± 0.32	n.s	n.d	n.s	*VEGF*	*n.d*	*n.d*	*n.s*	*n.d*	*n.s*	*n.d*	*n.s*
45							*LIX*	*1306.46 ± 543.03*	*1151.18 ± 601.73*	*n.s*	*604.37 ± 257.14*	*n.s*	*1211.56 ± 434.32*	*n.s*

## Data Availability

The data presented in this study are available from the authors on reasonable request.
